# Could the domestic cat play a significant role in the transmission of *Echinococcus multilocularis*? A study based on qPCR analysis of cat feces in a rural area in France

**DOI:** 10.1051/parasite/2016052

**Published:** 2016-10-14

**Authors:** Jenny Knapp, Benoît Combes, Gérald Umhang, Soufiane Aknouche, Laurence Millon

**Affiliations:** 1 Chrono-Environnement Laboratory, UMR UBFC/CNRS 6249 aff. INRA, University of Bourgogne Franche-Comté 25030 Besançon France; 2 Department of Parasitology-Mycology, University Hospital of Besançon 25030 Besançon France; 3 Entente for the Control of Zoonoses 54220 Malzéville France; 4 ANSES Nancy Laboratory for Rabies and Wildlife, National Reference Laboratory for Echinococcus spp., Wildlife Surveillance and Eco-Epidemiology Unit, Technopole Agricole et Vétérinaire 54220 Malzéville France

**Keywords:** *Echinococcus multilocularis*, *Felis catus domesticus*, Em-rrn qPCR, Host fecal PCR test, Parasite eggs

## Abstract

*Echinococcus multilocularis*, a cestode parasite responsible for alveolar echinococcosis in humans, is often reported in Europe. It involves red foxes, domestic dogs, and domestic and wild cats as definitive hosts. The parasite infects small mammals and accidentally humans as intermediate hosts and develops in a similar way to a tumor, usually in the liver. Domestic animals are suspected of playing a role in parasite transmission, but this is rarely proven. Moreover, the role of domestic cats is thought to be small, because of experimental studies showing incomplete development of the parasite observed in their intestines. In the present study, we investigated copro-sampling performed in a rural and highly endemic area in Eastern France, on carnivore feces (*n* = 150). From these samples, the parasite was detected and identified by DNA analysis using quantitative PCR targeting part of a mitochondrial gene (Em-qPCR). Taeniid eggs were isolated from positive-Em-qPCR samples by flotation, and species identification was confirmed by sequencing on DNA extracts. From a total of 43 copro-samples from cats, four tested positive for *E. multilocularis* by the Em-qPCR. In two of these, we found parasite eggs that were identified as *E. multilocularis*. This finding was confirmed by sequencing, while one dog stool out of 61 collected was found to be positive, no egg was detectable. At the same time, 34% of fox stools tested positive for the parasite. The present study challenges the current idea that cats are only of minor significance in the *E. multilocularis* life cycle.

## Abstract (fr)

*Echinococcus multilocularis*, un cestode parasite responsable de l’échinococcose alvéolaire chez l’homme est souvent mentionné en Europe. Il implique comme hôtes définitifs les renards roux, les chiens domestiques et les chats domestiques et sauvages. Le parasite implique, comme hôtes intermédiaires, des petits mammifères et accidentellement l’homme, et se développe de manière similaire à une tumeur, habituellement dans le foie. Les animaux domestiques sont suspectés de jouer un rôle dans la transmission du parasite, mais ce fait est rarement prouvé. De plus, le rôle des chats domestiques est connu pour être minime, en raison d’études expérimentales montrant un développement incomplet du parasite observé dans leurs intestins. Dans la présente étude nous avons examiné un échantillonnage coprologique réalisé dans une aire rurale hautement endémique dans l’Est de la France, sur des fèces de carnivores (*n* = 150). À partir de ces échantillons, le parasite a été détecté et identifié par analyse ADN en utilisant la PCR quantitative ciblant une partie d’un gène mitochondrial (qPCR-Em). Des œufs de Taeniidae ont été isolés à partir des échantillons positifs à la qPCR-Em par flottaison et l’identification spécifique a été confirmée par séquençage sur des extraits d’ADN. À partir d’un total de 43 copro-échantillons de chats, quatre ont été testés positifs pour *E. multilocularis* par la qPCR-Em. Pour deux d’entre eux, nous avons trouvé des œufs de parasites qui ont été identifiés comme étant *E. multilocularis*. Ce résultat a été confirmé par séquençage, alors qu’une selle de chien sur 61 collectées a été trouvée positive, mais aucun œuf n’a été détecté. Dans le même temps, 34 % des fèces de renards ont été testées positives pour le parasite. La présente étude interpelle sur l’idée actuelle que les chats ne joueraient qu’un rôle non significatif dans le cycle de vie d’*E. multilocularis*.

## Introduction

The tapeworm *Echinococcus multilocularis* is often described in Europe as involving wild definitive hosts (DHs), red foxes and increasingly raccoon dogs, domestic dogs and, more rarely, cats [4]. However, a wide range of intermediate hosts (IHs), such as small mammals, including voles [35], have been described. Aberrant IHs such as humans, monkeys, pigs, or even dogs have also been described [30]. The IHs are infected after consumption of parasite eggs, which are released into the environment from adult worms in carnivore stools. The parasite is the causative agent of alveolar echinococcosis in humans, one of the most challenging zoonoses in Europe [32], involving mass epidemiological surveillance [2].

The infection of humans by the parasite is likely to be due to multifactorial factors, e.g. consumption of wild berries, hunting activities, agricultural activities, having a dog, or through spending time outdoors [9, 18, 27]. In Europe, numerous field studies have been conducted on foxes to investigate the presence of *E. multilocularis*, both on necropsies [3, 13] and copro-samples [10, 19, 21], because of their role in parasite transmission and because of the high prevalence of *E. multilocularis* in these wild animals. By comparison, fewer studies have assessed the role of domestic dogs and cats in terms of the risk of *E. multilocularis* transmission. In an assessment of a copro-ELISA test in Switzerland, the parasite was described in 0.75% of dogs studied and 0.76% of cats from a cohort of 660 and 263 copro-samples, respectively, and confirmation was possible in about half of the positive dogs and cats after necropsy and/or PCR [8]. A French study conducted on 81 necropsied domestic cats in the Alps region highlighted a parasite prevalence of 2.4% [26]. No investigation has focused on the presence of eggs in the worms from these cats. A study based on routine veterinary parasite analysis conducted in Germany and other countries in Europe on copro-samples by egg flotation emphasized low *E. multilocularis* egg prevalence in dogs (0.24%) and cats (0.23%), but a real risk for humans was underlined [12]. In the Hokkaido area in Japan, the presence of *E. multilocularis* eggs in cat stools was described for the first time [24]. According to recent data, 6.9% of dog copro-samples in Swiss farmlands [16] have been described as positive after testing 402 dog feces by the flotation method. Moreover, another study was conducted on domestic and wild cats from North-Eastern France, in the Ardennes region, where a high prevalence of *E. multilocularis* [3] and human cases of alveolar echinococcosis has been reported [28]. One positive necropsied domestic cat (1/19 cats) and one wildcat (1/5 cats) were found to be positive, but only for immature worms. The evaluation of 321 feces from cats from the same area using qPCR [19] led to the detection of *E. multilocularis* DNA in 3.2% of stools but no *E. multilocularis* eggs were observed by flotation in the positive samples [34].

In these studies, the presence of eggs in cat stools was rarely observed or went undetected, because the diagnosis was often based only on the presence of adult worms in the intestines, and most of the studies focused on animals that visited the vet regularly, but investigations rarely involved blind sampling from fields. From experimental studies, the adult worms were shown to have lower egg production in cats than in foxes and dogs, and cats have been described as a poor host for the parasite [5, 17]. Therefore, contact with domestic animals such as dogs and cats has seldom been described in the literature as a reliable factor for parasite transmission [15, 18, 27]. However, due to the close and frequent contact these animals have with humans, they could potentially be important actors in parasite transmission to humans, even with low prevalence, especially in urban areas [22].

In the field of epidemiology, the recent advances in molecular biology on *E. multilocularis* copro-DNA samples [10, 19, 20, 33] enable us to investigate a large collection of samples using non-invasive sampling, and to identify the parasite and the host species simultaneously. This molecular approach has multiple advantages: it has high sensitivity, high specificity, is inexpensive, and could help improve studies on *E. multilocularis* transmission by domestic animals. The present study was carried out in order to assess the presence of *E. multilocularis* eggs in feces of cats and dogs from an endemic rural area observed as a snapshot, with initial screening by qPCR, research of *E. multilocularis* eggs by flotation combined with qPCR, and sequencing for confirmation.

## Materials and methods

### Copro-sampling

The study was conducted in an endemic area, in the Haute-Saône area of France, in a town located in the North-Eastern part of the country (47°35′27″ N, 6°24′56″ E). The surface area of the town was estimated at 5.78 km^2^. The human population density in the town was 25 inhabitants/km^2^ at the time of the study, and 146 inhabitants, from 2013 population census. The prevalence of *E. multilocularis* was recently assessed at 36% in red foxes [3] and about 50 human cases have been documented in Haute-Saône since 1984 (FrancEchino Data). Whole accessible copro-samples were collected in the course of one day, in January 2014. All the access roads to the town were investigated on both sides, at a 2 m distance from the road border. Morphological aspects of the copro-samples were noted. The samples were collected in an airtight plastic bag, with safety precautions (disposable chopsticks to manipulate the sample, placed into double plastic bags), and the sample was geolocated with a Global Positioning System (GPS) device. A total of 150 stool samples were collected. The samples were stored at −80 °C for seven days, in order to prevent any risk of infection, and stored at −20 °C before analysis. The distribution of stools from domestic animals was represented on a map using Quantum GIS software 1.8.0 (QGIS Development Team, 2012. QGIS Geographic Information System. Open Source Geospatial Foundation Project. http://qgis.osgeo.org).

### Copro-DNA extraction, qPCR protocols, and *E. multilocularis* diagnostic confirmation

The copro-DNA was purified using the QIAamp Fast DNA Stool kit, following the manufacturer’s recommendations (Qiagen, Hilden, Germany), on 0.5 g of sample, and eluted in 200 μL of the elution buffer provided. From the copro-DNA, a host fecal PCR test was performed to confirm the feces host identity. The test was carried out with real-time quantitative PCR (qPCR) as previously described, based on the amplification of a fragment of the *cytB* gene [20] (Vv-qPCR for *Vulpes vulpes*, Cf-qPCR for *Canis familiaris*, Fc-qPCR for *Felis catus*, Mm-qPCR for *Meles meles*, and Mf-qPCR for *Martes foina*). Copro-samples were tested for the presence of *E. multilocularis* DNA and quantification of it, based on the amplification of a part of the mitochondrial gene *rrnL* (Em-qPCR) [19]. The qPCR protocol was the same as that used by Knapp and co-workers [20], in a duplex qPCR, combining two hydrolysis probes with different fluorochromes and specific primer sets. The presence of inhibitor factors was tested using the Alea tool (Alea qPCR), previously developed [20], in a duplex qPCR (Em/Alea-qPCR, Vv/Cf-qPCR, and Fc/Alea-qPCR). All qPCR tests were performed twice in two different runs, so that four results were obtained for each sample. The host fecal test and *E. multilocularis* diagnosis were both confirmed by sequencing from long-qPCR products of host SEQ-PCR and Em SEQ-PCR [20], by using the Sanger sequencing method [29]. The sequences obtained were compared using the online genetic databases and the Basic Local Alignment Search Tool (BLAST) available on the National Center for Biotechnology Information (NCBI) website.

### Isolation and molecular identification of eggs from Em-qPCR-positive copro-samples

The copro-samples found to be positive with the Em-qPCR were tested for the presence of tapeworm eggs. A flotation protocol described by Dryden and co-workers was followed, based on the principle of differential specific gravity (SG) among parasite eggs, fecal debris, and the flotation solution, the latter having the greater SG [11]. The helminths SG was determined to range from 1.05 to 1.23 [6]. An NaCl solution (*d* = 1.2) was used as a flotation solution. Twenty milliliters of NaCl solution was added to 5 g of copro-sample and mixed for homogenization. The mixture was poured through a tea strainer to fill a new tube and formed a positive meniscus. A glass slide was applied to the meniscus for 20 min to collect eggs. The presence of tapeworm eggs was checked under an inverted microscope and manipulated with the DMI3000-B micromanipulator system (Leica Microsystems, Wetzlar, Germany), in order to isolate taeniid eggs and pool them for DNA purification. Furthermore, all slides were rinsed with Tris-EDTA pH 8.0 buffer solution for parasite DNA detection. The High Pure PCR Template Preparation kit (Roche, Mannheim, Germany) was used following the manufacturer’s recommendations to purify DNA. DNA was eluted in 100 μL of the elution buffer provided. Em-qPCR was performed on the purified egg DNA from flotation and SEQ-PCR was done for sequencing and confirmation of the identity of eggs as *E. multilocularis*. A comparison on the NCBI databases was performed as explained above.

## Results

### Copro-sampling and host fecal PCR test

**Figure 1.**
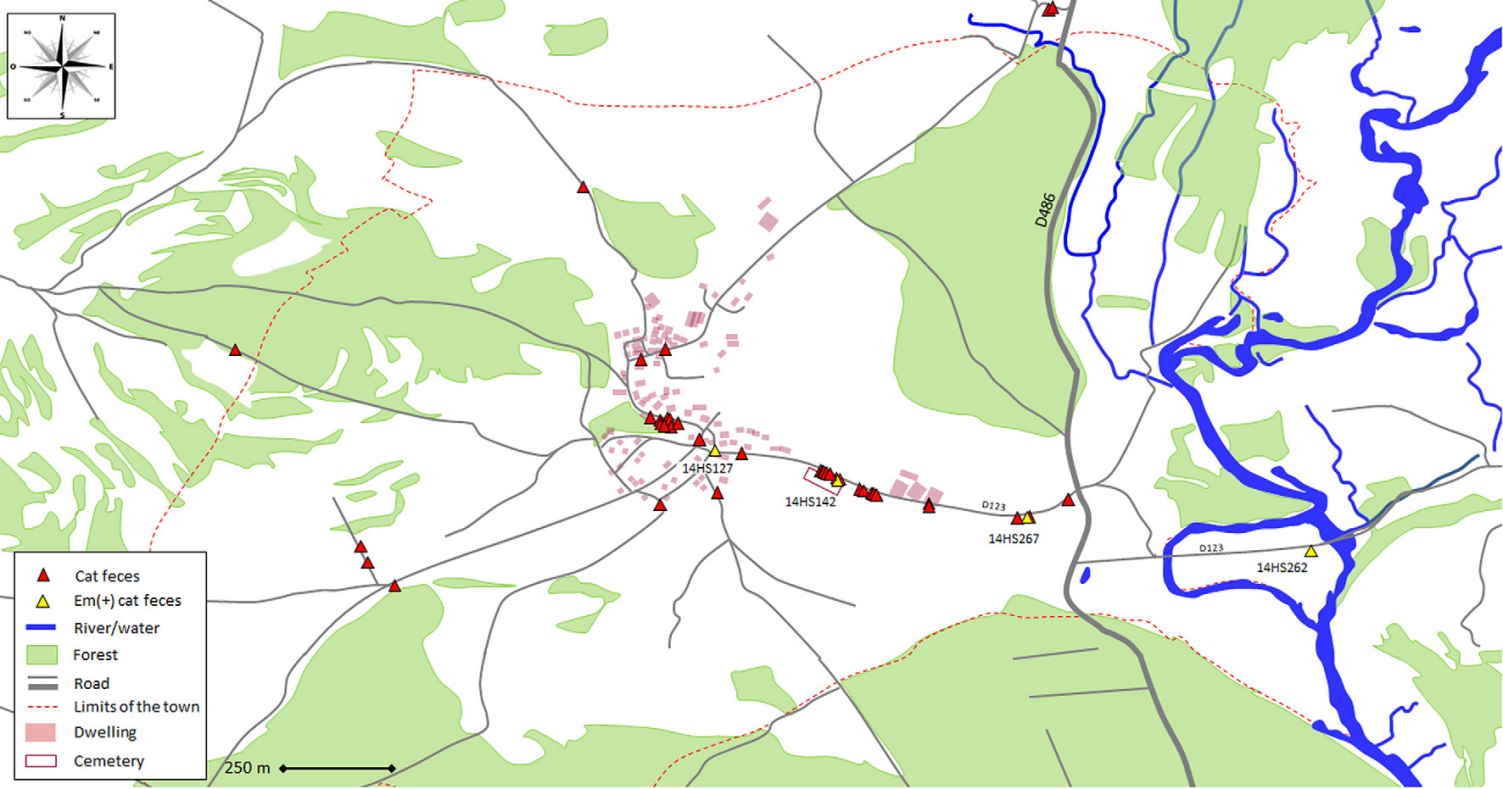
Map showing locations of cat feces, identified by host fecal PCR test and tested for *E. multilocularis* DNA presence by Em-qPCR in a rural town in France ((47°35′27″ N, 6°24′56″ E).

**Figure 2.**
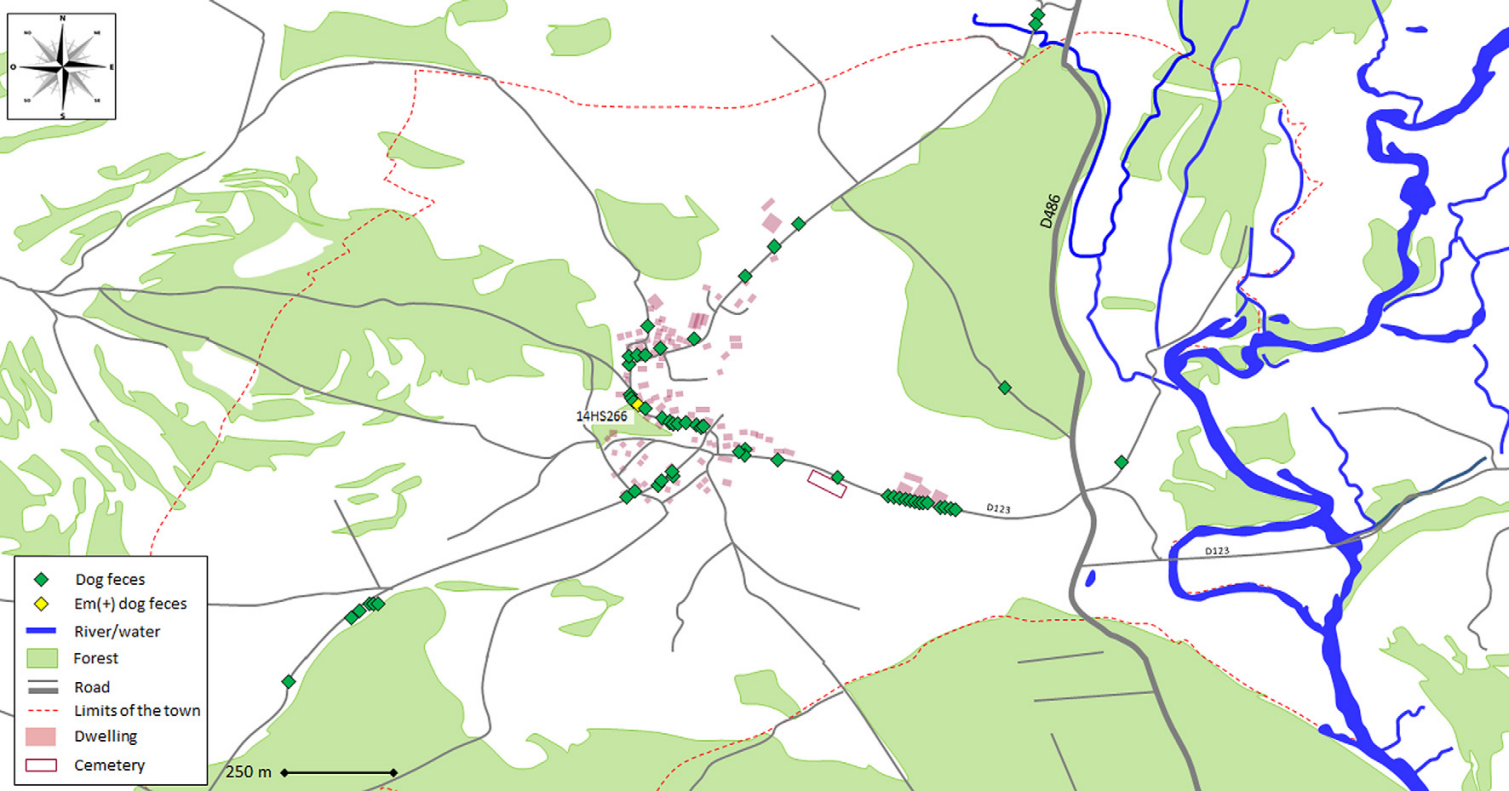
Map showing locations of dog feces, identified by host fecal PCR test and tested for *E. multilocularis* DNA presence by Em-qPCR in a rural town in France ((47°35′27″ N, 6°24′56″ E).

From the copro-sampling (*N* = 150), the host fecal PCR test enabled us to identify 43 feces from cats (29%), and 61 from dogs (41%). The feces were found in a wide area, from the center of the town all the way to the edge of the area studied (Figs. 1 and 2). The other feces were identified as belonging to red foxes for 35 samples (23%), 3 stone martens (2%) and 8 remained unidentified (5%). The Alea inhibitor presence test found one sample from the collection to be inhibited for the qPCRs and the host and parasite were classified as unidentifiable.

### *E. multilocularis* diagnosis

Four out of 43 cat feces presented a positive Em-qPCR (9.3%) on the same road in the village (Fig. 1). In two samples (14HS142 and 14HS267), Em-qPCR analyses were positive for the two duplicates in the two runs performed (4/4 positive analyses), with a Cq average of 35.2 and 38.4, respectively. For the 14HS142 and 14HS267 samples, the *E. multilocularis* diagnosis was confirmed on copro-DNA using the Em SEQ-PCR, with 100% identity with the referenced isolate GenBank Ref. KP941429.1. Taeniid eggs were observed under microscope after flotation and confirmed as *E. multilocularis* with the same nucleotide sequence obtained from the Em SEQ-PCR.

For the two other samples from cats (14HS127 and 14HS262), Em-qPCR were positive in only one replicate per run (1/4 positive analyses for each) with higher Cq (40.5 and 41.9, respectively). For these two latter samples with high Cq, no Em SEQ-PCR products could be obtained and no eggs were observed after flotation.

From the 61 dog feces, one sample was found to be positive for the Em-qPCR (1.6%) (Fig. 2), with only one positive replicate (sample 14HS266, Cq 39.9). For this sample, no Em SEQ-PCR products could be obtained and no eggs were observed after flotation.

For the 35 fox feces, 12 samples were found to be positive for the Em-qPCR (34%) and one positive for the unidentified samples. No positive qPCR was detected among stone marten feces.

Host identity was confirmed for the Em-qPCR-positive copro-samples in domestic animals by host SEQ-PCR product sequencing, with 100% identity with the reference isolate GenBank Ref. KU253483.1 for the cats and KU253532.1 for the dog.

## Discussion

The advantages of studies based on copro-DNA are numerous compared to necropsy. First, the collection of samples is easy to implement, a large number of samples can be stored and analyzed, the research on numerous DNA targets (e.g. parasites, metagenomic studies) is possible, and this sampling method is often considered more ethical by the public due to its gentle approach. Furthermore, in the case of *E. multilocularis* studies, an estimation of the risk of pathogen transmission to humans can be performed by observing eggs. Indeed, if *E. multilocularis* adult worms are observed in cats [8, 26, 34], the worms are unlikely to be able to produce eggs [17]. However, *E. multilocularis* has previously been observed in a highly endemic area in France, involving domestic mice and probably domestic animals, harboring fertile worms [25]. A domestic cycle can then occur.

In the present study, a relatively high prevalence of *E. multilocularis* was observed from cat stools (9.3%) in comparison to previous studies, whereas the prevalence in dogs (1.6%) is consistent with the literature [12]. In cats, this could be due to the relatively small number of samples tested or the possibility that more than one fecal sample originated from the same infected cat. Individual genotyping should be performed to discriminate between the different animals excreting in towns with the current molecular tools available. Indeed, Menotti-Raymond and co-workers identified 11 tetranucleotide short tandem repeat (STR) loci in domestic cats for the genetic identification of cats, even in inbred cat populations [23]. Nevertheless, the present survey highlights an environmental risk of infection by *E. multilocularis* with the contaminating sources spread by hosts. In cats and dogs, the samples with the highest qPCR Cq values (Cq ≥ 39.9) were not confirmed by sequencing on copro-DNA or on flotation products. These results correspond to picograms of DNA detected in the 0.5 g of the copro-samples [20]. From a previous study, for the present Em-qPCR, it was described that beyond 38 cycles of PCR, DNA was considered to be detectable but not quantifiable [20]. Moreover, although small quantities of DNA can be detected by qPCR, this represents less than the DNA contained in one egg [19]. Indeed, it is possible that parasite DNA may be detected in copro-samples after ingestion of contaminated prey [34]. Moreover, the DNA from released worms could also be detected. The information about the presence of the parasite in this way is nevertheless interesting. On the other hand, one could consider these samples as false-negatives as only one replicate was positive in Em-qPCR. First, the SEQ-PCR [20] enabled us to confirm positive copro-samples; second the flotation step and sequencing permitted us to confirm the presence of *E. multilocularis* eggs. The possible risk of contamination to intermediate hosts could be assessed, rather than simply the detection of DNA traces without excreted eggs. However, if eggs can be observed in cat feces, a final experiment should be conducted on the viability and infectiousness of these eggs, especially as previous experimental infection of cats by *E. multilocularis* has only resulted in non-infectious eggs in the mouse [17]. However, there is a high risk of infection for the handler in this experiment and it should therefore be performed with caution.

The involvement of domestic animals such as cats and dogs in parasite spreading is also worrying when animals travel, with no preventive de-worming, veterinary advice, or border controls. Countries that are *E. multilocularis*-free are particularly concerned about importing the parasite into their countries by domestic dogs and cats [7, 14, 31]. Indeed, a high risk of contamination in dogs traveling from Britain to Germany has been found, emphasizing the importance of praziquantel treatment prior to travel for domestic animals [31]. Moreover, information provided by vets in different European countries to people intending to travel with pets in Europe was estimated to be insufficient [7]. As far as transmission to humans is concerned, the level of knowledge of alveolar echinococcosis among primary care physicians and pharmacists in France was also assessed [1] and remains poor even in endemic regions, especially relating to the question of sources of contamination.

Our study emphasized the potential role of cats in transmitting *E. multilocularis* to humans, with the detection of eggs in cat stools. Not only local public health campaigns, but also awareness programs on the risks to human and animal health, especially to promote the de-worming of pets and the practice of basic hygiene rules when handling soil or animals, should be conducted.

## References

[1] Bourgeois B, Marguet P, Gbaguidi-Haore H, Knapp J, Said-Ali Z, Demonmerot F, Bresson-Hadni S, Millon L, Bellanger AP. 2015 Alveolar echinococcosis: How knowledgeable are primary care physicians and pharmacists in the Franche-Comté region of France? Acta Parasitologica, 60(4), 682–690. 26408591 10.1515/ap-2015-0097

[2] Charbonnier A, Knapp J, Demonmerot F, Bresson-Hadni S, Raoul F, Grenouillet F, Millon L, Vuitton DA, Damy S. 2014 A new data management system for the French National Registry of human alveolar echinococcosis cases. Parasite, 21, 70. 25526544 10.1051/parasite/2014075 PMC4271653

[3] Combes B, Comte S, Raton V, Raoul F, Boué F, Umhang G, Favier S, Dunoyer C, Woronoff N, Giraudoux P. 2012 Westward spread of *Echinococcus multilocularis* in foxes, France, 2005–2010. Emerging Infectious Diseases, 18(12), 2059–2062. 23171707 10.3201/eid1812.120219 PMC3557902

[4] Conraths FJ, Deplazes P. 2015 *Echinococcus multilocularis*: Epidemiology, surveillance and state-of-the-art diagnostics from a veterinary public health perspective. Veterinary Parasitology, 213(3–4), 149–161. 26298509 10.1016/j.vetpar.2015.07.027

[5] Crellin JR, Marchiondo AA, Andersen FL. 1981 Comparison of suitability of dogs and cats as hosts of *Echinococcus multilocularis*. American Journal of Veterinary Research, 42(11), 1980–1981. 7337294

[6] David ED, Lindquist WD. 1982 Determination of the specific gravity of certain helminth eggs using sucrose density gradient centrifugation. Journal of Parasitology, 68(5), 916–919. 6890102

[7] Davidson RK, Robertson LJ. 2012 European pet travel: misleading information from veterinarians and government agencies. Zoonoses Public Health, 59(8), 575–583. 22639949 10.1111/j.1863-2378.2012.01499.x

[8] Deplazes P, Alther P, Tanner I, Thompson RC, Eckert J. 1999 *Echinococcus multilocularis* coproantigen detection by enzyme-linked immunosorbent assay in fox, dog, and cat populations. Journal of Parasitology, 85(1), 115–121. 10207375

[9] Deplazes P, Hegglin D, Gloor S, Romig T. 2004 Wilderness in the city: the urbanization of *Echinococcus multilocularis*. Trends in Parasitology, 20(2), 77–84. 14747021 10.1016/j.pt.2003.11.011

[10] Dinkel A, Kern S, Brinker A, Oehme R, Vaniscotte A, Giraudoux P, Mackenstedt U, Romig T. 2011 A real-time multiplex-nested PCR system for coprological diagnosis of *Echinococcus multilocularis* and host species. Parasitology Research, 109(2), 493–498. 21327991 10.1007/s00436-011-2272-0

[11] Dryden MW, Payne PA, Ridley R, Smith V. 2005 Comparison of common fecal flotation techniques for the recovery of parasite eggs and oocysts. Veterinary Therapeutics: Research in Applied Veterinary Medicine, 6(1), 15–28. 15906267

[12] Dyachenko V, Pantchev N, Gawlowska S, Vrhovec MG, Bauer C. 2008 *Echinococcus multilocularis* infections in domestic dogs and cats from Germany and other European countries. Veterinary Parasitology, 157(3–4), 244–253. 18819752 10.1016/j.vetpar.2008.07.030

[13] Enemark HL, Al-Sabi MN, Knapp J, Staahl M, Chríel M. 2013 Detection of a high-endemic focus of *Echinococcus multilocularis* in red foxes in southern Denmark, January 2013. Euro Surveillance, 18(10), 20420. 23515060 10.2807/ese.18.10.20420-en

[14] Goodfellow M, Shaw S, Morgan E. 2006 Imported disease of dogs and cats exotic to Ireland: *Echinococcus multilocularis*. Irish Veterinary Journal, 59(4), 214–216. 21851681 10.1186/2046-0481-59-4-214 PMC3113889

[15] Gottstein B, Saucy F, Deplazes P, Reichen J, Demierre G, Busato A, Zuercher C, Pugin P. 2001 Is high prevalence of *Echinococcus multilocularis* in wild and domestic animals associated with disease incidence in humans? Emerging Infectious Diseases, 7(3), 408–412. 11384517 10.3201/eid0703.010307 PMC2631798

[16] Hauser M, Basso W, Deplazes P. 2015 Dog and fox faecal contamination of farmland. Schweizer Archiv für Tierheilkunde, 157(8), 449–455. 26753365 10.17236/sat00030

[17] Kapel CMO, Torgerson PR, Thompson RCA, Deplazes P. 2006 Reproductive potential of *Echinococcus multilocularis* in experimentally infected foxes, dogs, raccoon dogs and cats. International Journal for Parasitology, 36(1), 79–86. 16199043 10.1016/j.ijpara.2005.08.012

[18] Kern P, Bardonnet K, Renner E, Auer H, Pawlowski Z, Ammann RW, Vuitton DA, Kern P, European Echinococcosis Registry. 2003 European echinococcosis registry: human alveolar echinococcosis, Europe, 1982–2000. Emerging Infectious Diseases, 9(3), 343–349. 12643830 10.3201/eid0903.020341 PMC2958541

[19] Knapp J, Millon L, Mouzon L, Umhang G, Raoul F, Ali ZS, Combes B, Comte S, Gbaguidi-Haore H, Grenouillet F, Giraudoux P. 2014 Real time PCR to detect the environmental faecal contamination by *Echinococcus multilocularis* from red fox stools. Veterinary Parasitology, 201(1–2), 40–47. 24484767 10.1016/j.vetpar.2013.12.023

[20] Knapp J, Umhang G, Poulle ML, Millon L. 2016 Development of a real-time PCR for a sensitive one-step copro-diagnosis allowing both the identification of carnivore feces and the detection of *Toxocara* spp. and *Echinococcus multilocularis*. Applied and Environmental Microbiology, 82(10), 2950–2958. 26969697 10.1128/AEM.03467-15 PMC4959075

[21] Learmount J, Zimmer IA, Conyers C, Boughtflower VD, Morgan CP, Smith GC. 2012 A diagnostic study of *Echinococcus multilocularis* in red foxes (*Vulpes vulpes*) from Great Britain. Veterinary Parasitology, 190(3–4), 447–453. 22840643 10.1016/j.vetpar.2012.07.003

[22] Massolo A, Liccioli S, Budke C, Klein C. 2014 *Echinococcus multilocularis* in North America: the great unknown. Parasite, 21, 73. 25531581 10.1051/parasite/2014069 PMC4273702

[23] Menotti-Raymond MA, David VA, Wachter LL, Butler JM, O’Brien SJ. 2005 An STR forensic typing system for genetic individualization of domestic cat (*Felis catus*) samples. Journal of Forensic Sciences, 50(5), 1061–1070. 16225210

[24] Nonaka N, Hirokawa H, Inoue T, Nakao R, Ganzorig S, Kobayashi F, Inagaki M, Egoshi K, Kamiya M, Oku Y. 2008 The first instance of a cat excreting *Echinococcus multilocularis* eggs in Japan. Parasitology International, 57(4), 519–520. 18664390 10.1016/j.parint.2008.07.001

[25] Pétavy AF, Deblock S, Walbaum S. 1990 The house mouse: a potential intermediate host for *Echinococcus multilocularis* in France. Transactions of the Royal Society of Tropical Medicine and Hygiene, 84(4), 571–572. 2091354 10.1016/0035-9203(90)90044-f

[26] Pétavy AF, Tenora F, Deblock S, Sergent V. 2000 *Echinococcus multilocularis* in domestic cats in France. A potential risk factor for alveolar hydatid disease contamination in humans. Veterinary Parasitology, 87(2–3), 151–156. 10622606 10.1016/s0304-4017(99)00181-8

[27] Piarroux M, Piarroux R, Knapp J, Bardonnet K, Dumortier J, Watelet J, Gerard A, Beytout J, Abergel A, Bresson-Hadni S, Gaudart J, FrancEchino Surveillance Network. 2013 Populations at risk for alveolar echinococcosis, France. Emerging Infectious Diseases, 19(5), 721–728. 23647623 10.3201/eid1905.120867 PMC3647496

[28] Piarroux M, Gaudart J, Bresson-Hadni S, Bardonnet K, Faucher B, Grenouillet F, Knapp J, Dumortier J, Watelet J, Gerard A, Beytout J, Abergel A, Wallon M, Vuitton DA, Piarroux R, FrancEchino network. 2015 Landscape and climatic characteristics associated with human alveolar echinococcosis in France, 1982 to 2007. Euro Surveillance, 20(18), 21118. 25990231 10.2807/1560-7917.es2015.20.18.21118

[29] Sanger F, Nicklen S, Coulson AR. 1992 DNA sequencing with chain-terminating inhibitors, 1977. Biotechnology, 24, 104–108. 1422003

[30] Thompson R, McManus D. 2002 Aetiology: parasites and life-cycles. Manual WHO/OIE on Echinococcosis in humans and animals: a public health problem of global concern, Chapter 1, p. 9.

[31] Torgerson PR, Craig PS. 2009 Risk assessment of importation of dogs infected with *Echinococcus multilocularis* into the UK. Veterinary Record, 165(13), 366–368. 19783849 10.1136/vr.165.13.366

[32] Torgerson PR, Keller K, Magnotta M, Ragland N. 2010 The global burden of alveolar echinococcosis. PLoS Neglected Tropical Diseases, 4(6), e722. 20582310 10.1371/journal.pntd.0000722 PMC2889826

[33] Trachsel D, Deplazes P, Mathis A. 2007 Identification of taeniid eggs in the faeces from carnivores based on multiplex PCR using targets in mitochondrial DNA. Parasitology, 134(Pt 6), 911–920. 17288631 10.1017/S0031182007002235

[34] Umhang G, Forin-Wiart M-A, Hormaz V, Caillot C, Boucher J-M, Poulle M-L, Franck B. 2015 *Echinococcus multilocularis* detection in the intestines and feces of free-ranging domestic cats (*Felis s. catus*) and European wildcats (*Felis s. silvestris*) from northeastern France. Veterinary Parasitology, 214(1–2), 75–79. 26206606 10.1016/j.vetpar.2015.06.006

[35] Vuitton DA, Zhou H, Bresson-Hadni S, Wang Q, Piarroux M, Raoul F, Giraudoux P. 2003 Epidemiology of alveolar echinococcosis with particular reference to China and Europe. Parasitology, 127(Suppl), S87–S107. 15027607

